# Applications of chemically modified screen-printed electrodes in food analysis and quality monitoring: a review

**DOI:** 10.1039/d4ra02470b

**Published:** 2024-09-02

**Authors:** Kavitha Kamalasekaran, Ashok K. Sundramoorthy

**Affiliations:** a Department of Chemistry, Velammal Engineering College Chennai 600066 Tamil Nadu India; b Centre for Nano-Biosensors, Department of Prosthodontics and Materials Science, Saveetha Dental College and Hospitals, Saveetha Institute of Medical and Technical Sciences Chennai 600077 Tamil Nadu India ashok.sundramoorthy@gmail.com

## Abstract

Food analysis and food quality monitoring are vital aspects of the food industry, ensuring the safety and authenticity of various food products, from packaged goods to fast food. In this comprehensive review, we explore the applications of chemically modified Screen-Printed Electrodes (SPEs) in these critical domains. SPEs have become extremely useful devices for ensuring food safety and quality assessment because of their adaptability, affordability, and convenience of use. The Introduction opens the evaluation, that covers a wide spectrum of foods, encompassing packaged, junk food, and food quality concerns. This sets the stage for a detailed exploration of chemically modified SPEs, including their nature, types, utilization, and the advantages they offer in the context of food analysis. Subsequently, the review delves into the multitude applications of SPEs in food analysis, ranging from the detection of microorganisms such as bacteria and fungi, which are significant indicators of food spoilage and safety, to the identification of pesticide residues, food colorants, chemicals, toxins, and antibiotics. Furthermore, chemically modified SPEs have proven to be invaluable in the quantification of metal ions and vitamins in various food matrices, shedding light on nutritional content and quality.

## Abstract 2

Disposable adlayer modified screen-printed electrodes have become extremely useful devices for ensuring food safety and quality assessment because of their adaptability, affordability, and convenience of use.

## Graphical abstract

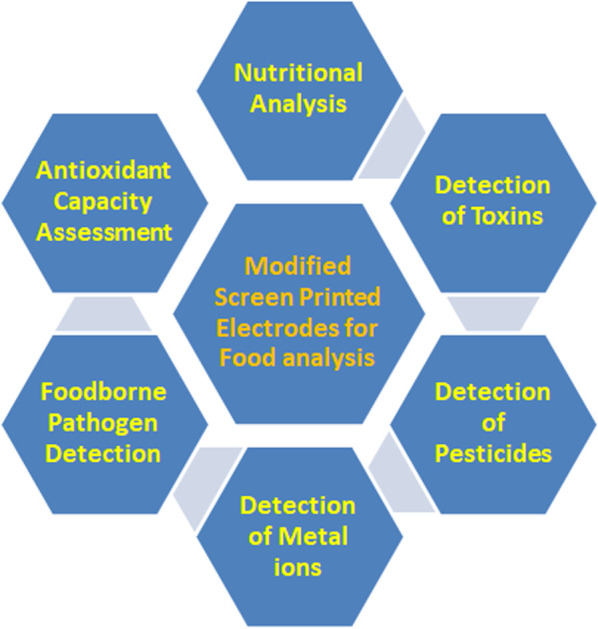

## 1. Introduction

The modern world's diverse culinary landscape encompasses a wide array of foods, from meticulously crafted delicacies to conveniently packaged meals, and from health-conscious, wholesome options to the irresistible allure of junk food. Food, an essential element of human existence, has evolved into a complex global industry, with products that cater to every palate, lifestyle, and dietary preference. In the midst of this remarkable diversity, the universal concern for food quality and safety resonates across all corners of the globe.^1^ This concern reaches far and wide, extending beyond the boundaries of cuisine. It encapsulates the nutritional integrity of the groceries we purchase, the hygiene of the meals we consume, and the authenticity of the flavours we savour. The assurance of food quality is not merely a matter of taste; it is a cornerstone of public health and well-being. In a world characterized by rapid urbanization, food globalization, and an ever-accelerating pace of life, ensuring the quality of the foods we consume is a pressing imperative.^2,3^

Packaged foods, emblematic of convenience and a busy lifestyle, line the shelves of supermarkets and convenience stores. These products bear the promise of enduring freshness, standardized quality, and detailed nutritional information.^4^ These foods represent a captivating interplay of chemistry within the realm of modern nutrition. These products, an outcome of meticulous food processing techniques, owe their sensory allure to a web of intricate chemical reactions.^5^ The inclusion of various chemical additives, such as antioxidants and emulsifiers, adds a layer of complexity, impacting shelf life and sensory properties.^6^ Ensuring the integrity of packaged foods, from the labelling to the contents within, is an ongoing pursuit.

Junk food, often an irresistible indulgence, captures the essence of guilty pleasures. These highly processed and indulgent treats tantalize the taste buds while raising concerns about excessive sugars, fats, and artificial additives.^7^ Monitoring the quality of junk food is essential, given its association with obesity, chronic diseases, and nutritional imbalances.^8^

Junk foods, ubiquitous in modern diets, possess a complex chemistry. These are rich in unhealthy fats, sugars, and salts, they undergo processes like Maillard reactions and lipid oxidation. These reactions contribute to the characteristic flavors, textures, and colors of these products. Nevertheless, consuming these items in excess can result in the development of advanced glycation end products (AGEs), which have been connected to long-term health problems. Understanding the chemistry of junk foods is crucial for addressing their impact on public health and nutrition.

Food quality concerns extend to the domains of foodborne pathogens, contaminants, and adulterants. Microorganisms, such as bacteria and fungi, can thrive in food products, causing spoilage or, more critically, posing health risks.^9,10^ Pesticide residues and chemical contaminants can find their way into the food supply chain, necessitating rigorous monitoring. The presence of artificial colorants, flavor enhancers, or adulterants may compromise the authenticity and safety of various food items. Moreover, toxins and antibiotics, when present in edibles, demand swift detection to prevent health repercussions.

As the food industry navigates these complexities, innovative solutions are essential for safeguarding food quality and ensuring consumer confidence. One such innovation is the utilization of Chemically Modified Screen-Printed Electrodes (SPEs) in the analysis of various food components.^11,12^ SPEs, characterized by their versatility, cost-effectiveness, and ease of use, have been emerged as a powerful tool for food quality assessment and safety assurance. This review explores the multifaceted applications of chemically modified SPEs in the context of food analysis, with a focus on their ability to address a spectrum of food quality issues. Through this exploration, our goal is to provide insight into the critical role that SPEs play in contemporary food science and their potential benefits which will be beneficial for food safety and quality in the years to come.

The absence of literature reviews on chemically modified Screen-Printed Electrodes (SPEs) in food analysis underscores a crucial gap in understanding. As technology advances, the scope for SPEs extends into cutting-edge domains like artificial intelligence (AI), blockchain, multimodal sensors, and wireless connectivity. Integrating SPEs with these latest technologies holds immense potential for revolutionizing food analysis methods. The novelty of this paper lies in its comprehensive review, exploring the uncharted territory of SPEs in conjunction with advanced technologies, providing insights not only into current applications but also shedding light on the future trends and transformative impact of chemically modified SPEs in the realm of advanced technologies.

## 2. Importance of screen-printed electrodes (SPEs)

**Fig. 1:**
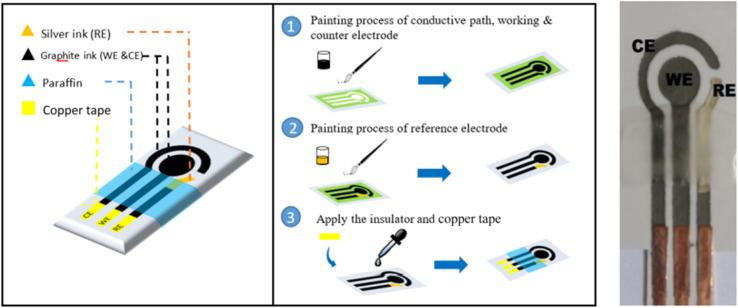
The simple fabrication of various kinds of SPE for various applications. Three electrodes (working, counter and reference) were printed.^13^ Similarly, gold, platinum and graphite coated working SPEs are also can be prepared with three electrodes configurations.

Screen-printed electrodes (SPEs) are a fundamental component of the field of electrochemistry, enabling precise and cost-effective electrochemical measurements. These electrodes are manufactured through a versatile screen-printing process, which allows for the creation of customized electrode designs on non-conductive substrates. SPEs consist of working, counter, and reference electrodes, serving as essential tools in applications ranging from environmental monitoring to clinical diagnostics (Fig. 1).^13^ Their affordability and ease of mass production have made SPEs an invaluable resource for researchers and professionals, offering a practical and accessible means of studying electrochemical reactions and quantifying various analytes with precision and convenience. The standard procedures for food analysis are often carried out in centralized laboratories, with lengthy analysis timeframes and intricate equipment. In this regard, sensors built on screen-printed electrodes (SPEs) have become more and more significant due to their beneficial attributes, like mobility and ease of use, which enable quick analysis in situations where it's needed. With an emphasis on the identification of important metal toxins, pathogens, bacteria and fungi, this review offers a thorough overview of SPE-based sensors for the assessment of food safety and quality analysis.

### 2.1 Description and processes including types and modifications of SPEs

The devices known as SPEs have become more and more popular in the last several years because of their portability, affordability, and simplicity of use. There are several types of SPEs, each designed for specific applications. SPEs are set up as an entire miniaturized electrochemical cell, usually with the working electrode (WE), the counter electrode (CE), and the reference electrode (RE).^14,15^ An image or pattern can be printed onto materials like paper, cloth, or polymer using the widely used screen printing technique. The process involves applying conductive ink to the surface by means of a stencil, also known as a screen. In light of this, the name of the method is screen-printed.^16^ Carbon-based screen-printed electrodes are prepared using screen-printing processes, carbon inks (including variables such graphite or carbon nanotubes) are usually printed on a substrate to create these electrodes.^17^ For the fabrication of SPEs, graphite ink, CNT ink, and Gr ink are frequently utilized.^18^ The most exciting advancements in electrochemical biosensors includes DNA sensors,^19^ aptasensors,^20^ immunosensors,^21^ and enzymatic biosensors.^22^ Non-biological materials used in thick-film technology include substrates like alumina, ceramics, PVC, gold, iron, *etc.*, and the electrode's conducting pad, which is made of platinum or another metal paste or carbon ink or paste. Traditionally, the thick-film biosensor construction employed biorecognition elements such as cells, DNA, RNA, enzymes, and antibodies. Screen-printed electrode structures are also frequently incorporated with mediators (*e.g.*, Meldola blue, Prussian blue), cofactors (*e.g.*, reduced form of nicotinamide adenine dinucleotide (NADH), pyrroloquinoline quinone (PQQ)), stabilizers, immobilization matrixes and/or additives (*e.g.*, cellulose acetate, Nafion), and cross-linkers (*e.g.*, glutaraldehyde) to enhance the biosensor's sensitivity, selectivity, stability, and repeatability.^23^

The ceramic substrate-based screen-printed electrodes are durable and perfect for conducting electrochemical studies in non-aqueous electrolytes or at high temperatures.^24^ Using well-known industrial printers, screen printed electrodes are created by depositing a mixture of layers onto a smooth surface. In terms of electrode design versatility, material compatibility, and customization, screen printing provides mass-producible, inexpensive, and highly achievable sensors. Conducting materials including carbon, graphene, and metals (silver, gold, platinum, *etc.*) are typically found in ink. Changes in the composition of screen-printed carbon inks have a significant impact on the reactivity of electron transport and the analytical performance of SPEs.^25^ In SPEs, conductive gold and silver inks are also used. The functional electrode is printed using carbon or gold inks, while the conductive track is printed using silver ink.^26^ For decades, a large number of researchers have been investigating new materials that can be utilized to modify the composition of printing inks. These materials fall into four basic categories: carbon-based (graphene, carbon nanotubes, carbon black), organic (chitosan and organic conducting polymers), inorganic (gold, silver), and composite modifiers. The primary application of this approach is a nanoparticle-based screen-printed electrode modification. Numerous functional nanomaterials and synthetic detection elements have been successfully added to it.^27^

SPEs are the basis of electrochemical (bio) sensors that are gaining popularity as analytical tools for food analysis because SPEs offer several benefits that give these kinds of sensors the crucial qualities of the best biosensors such as simplicity of use, affordability, and mobility.^28,29^ Thus, the development of screen-printed technology has played a major role in the shift from heavy, conventional electrochemical cells to tiny, portable electrodes that may be used for on-site studies.^30,31^

The type of SPE chosen will rely on the particular application and target analyte. Obviously, researchers and scientists have to select the most appropriate type of SPE to meet their unique requirements, taking into various factors such as electrode material, size, and the chemical or biological modifications needed for their experiments or measurements.

## 3. Diverse applications of SPEs in various fields

SPEs have a wide range of applications across various fields due to their versatility and affordability. The use of SPEs as transducers in electrochemical biosensors is a rapidly developing field with great promise. Their exceptional qualities, such as their portability, low volume loading, and disposability, make them perfect for on-site detection in the environmental, clinical, and food sectors. Additionally, the wide variety of electrochemical biosensor designs is expanded by their subsequent modification using functional proteins.^32,33^

SPEs are employed to detect and quantify environmental pollutants, heavy metals, and other substances in water, soil, and air. They contribute to environmental monitoring efforts and help assess the quality of natural resources.^34^ A lot of conventional air pollution analyzers are bulky, heavy, and very costly.^35^ Low-cost disposable sensors are utilized for monitoring soil and water contaminants, although they are not frequently used for gas sensing.^36^ Electroanalysis and SPEs are a great low-cost substitute for *in situ* environmental monitoring.^37^ Conventional methods for monitoring pollutants and other environmental factors have low limit of detection (LODs) of about parts per billion (ppb); they are also very sensitive, specific, and repeatable. However, they do require expensive, centralized laboratories with high power consumption, sample preparation, and highly skilled experimentalists.^38^ In the field of healthcare, SPEs play a crucial role in medical diagnostics. They are used for blood glucose monitoring, cholesterol analysis, and the detection of biomarkers related to diseases, making them essential for point-of-care testing.^39–42^ Development of inexpensive, compact, and user-friendly analytical instruments that deliver quantitative information quickly and easily on-site is clearly of interest. Due to their great potential, these devices can be applied in a wide range of sectors, including environmental monitoring,^43–46^ food analysis and quality control,^28,47,48^ and clinical and biomedical applications.^49–53^ SPEs are vital for a wide range of applications, from basic research to real-world applications due to their adaptability and accessibility.

## 4. Characteristics and advantages of SPE's

The success of SPEs as transducers in electroanalytical devices make sense when one considers all of its positive qualities, chief among them being their disposability when paired with precision. Although SPEs are now widely used for food analysis and quality control, paper was demonstrated for two decades to be an innovative substrate for the development of promising analytical devices. It enables flexibility in terms of electrode arrangement, material compatibility, and alterations while providing affordable and highly reproducible sensors and biosensors.^54–57^ The most significant characteristic of SPE sensors are their low cost, large production capacity, and tiny microelectronic size, which enable the sample volume needed for analysis to be as small as a few microliters. The salient characteristic of this SPE is that it minimizes the overall size of the electrochemical sensor device while permitting the monitoring instrument to be attached to the portable instruments.^58–62^ Their diverse applications versatility, portability, customization and cost effectiveness made as an absolute alternative to traditional electrochemical sensors.

## 5. SPE's in food analysis

In order to reduce food waste and avoid foodborne diseases, SPE-based sensors are used as complementary analytical tools to traditional approaches in the management of food spoilage. This allows for quick screening at any stage of the food supply chain. SPEs have emerged as invaluable tools in the realm of food analysis, providing an efficient and versatile platform for assessing food quality, safety, and nutritional content. In order to assess food safety and quality, this review offers a thorough overview of SPE-based sensors. It focuses on identifying significant contaminants in food samples, such as metal ions, toxins, pesticides, and food infectious agents. Following the discussion of food contaminants, a detailed description is given on the evaluation of food samples' antioxidant capacity, analysis of vital nutrients with the identification of food quality, and detection of food safety requirements.

### 5.1 Detection of food contaminants

#### 5.1.1. Detection of toxins

Numerous electrochemical paper-based sensors have been created since they were initially introduced a decade ago in order to detect toxins in food. Food poisoning and other foodborne illnesses are frequently brought on by these pollutants. Furthermore, a number of proteins in food can cause hypersensitive reactions in some individuals. Consequently, quick food material analysis *via* electrochemical paper-based sensors may offer an extra degree of security to stop consumers from consuming food that is contaminated. The uses of the sensors for identifying each class of contaminants will be covered in more detail in the sections that follow.

SPEs are employed to detect toxins and quantify contaminants in food products, including heavy metals, mycotoxins, and pesticide residues. These contaminants can pose health risks, and SPEs provide a rapid and cost-effective means of analysis. In the study conducted by Jigyasa *et al.*^63^ an electrochemical nitrite biosensor was developed using synthesised alkali metal (Na/K) doped graphitic carbon nitride (g-C_3_N_4_). The Na-infused g-C_3_N_4_ (Na–CN-300) exhibited impressive electrochemical performance for nitrite detection, exhibiting a low detection limit of 1.9 μM and a linear response in the concentration range of 10 μM to 2 mM. The sensor displayed excellent selectivity, sensitivity, and stability over 30 days, making it a helpful tool for nitrite measurement in water and food samples. The study offers a novel and facile method for synthesizing doped graphitic nitride materials and utilizing them for electrochemical sensing applications.

In another research, Orduz *et al.* presents a new amperometric sensor for triclosan detection utilising a screen-printed carbon nanotube electrode upgraded with Guinea grass peroxidase (GGP).^64^ The GGP-modified electrode demonstrated enhanced electrochemical response to triclosan compared to the bare electrode. It exhibited high sensitivity and a low detection limit of 3 μM for triclosan. The system integrates carbon nanotubes and GGP to create a potential tool for environmental analysis and food quality control. This electrochemical detection system offers promising applications for *in situ* analysis of environmental samples. An additional investigation into the label-free detection of Aflatoxin B1 (AFB1) in food samples using an aptasensing device built magnetically. The micro electrolytic cell is a disposable screen-printed carbon electrode (SPCE) covered in a polydimethylsiloxane (PDMS) layer. The device employs thiolated aptamers immobilized on Fe_3_O_4_@Au magnetic beads, put together on the SPCE working electrode within seconds. The sensor demonstrates a wide range for AFB1 detection (20 pg mL^−1^ to 50 ng mL^−1^) with a low detection limit (15 pg mL^−1^). It offers a simple, economical, and highly sensitive solution for food safety quality control and has the potential to be adapted for other target molecules if specific aptamers are available.^65^

For the voltammetric measurement of Roxarsone (ROX) in chicken purge and river water samples, a lab-made screen-printed electrode based on poly(ethylene terephthalate) (PET) substrate modified with a hybrid film containing gold nanoparticles-decorated graphene (AuNPs-GRA/PET-SPE) was used. In diluted river water and chicken purge samples, the technique produced a limit of detection of 60 nM and 97 nM for ROX, respectively.^66^ In order to detect foodborne pathogens with high sensitivity and selectivity without the need for washing, an electrochemical biosensor chip for signal amplification was created. This chip integrates methyl blue (MB) as the hybridization redox indicator and loop-mediated isothermal amplification (LAMP), which is based on *in situ* nucleic acid amplification. The SPE used in the electrochemical biosensor chip design was altered with gold nanoparticles (Au NPs) and encased in a polydimethylsiloxane membrane to create a microcell.^67^ Bacterial diseases such as listeriosis can cause meningitis, gastroenteritis, infections in pregnant women, and other symptoms. In 25–30% of cases, listeriosis is fatal.^68^ The tungsten sulphide (WS_2_) nanostructure was added to a screen-printed carbon electrode (SPCE) to create the aptasensor, which was then immobilized using an aptamer. In this work, a sophisticated electrochemical biosensor for the detection of *Salmonella enterica* serovar typhimurium (*S. typhimurium*) is presented. The invention uses mercaptoacetic acid (MAA)-induced self-assembled monolayers (SAM) production to immobilize anti-*Salmonella* antibodies on a gold (Au) electrode. Its effectiveness in artificially contaminated food and water samples was in line with conventional techniques, highlighting it's reliability in real-world situations.^69^

#### 5.1.2. Detection of pesticides

Monitoring pesticides is crucial for food quality due to their adverse effects on human health and ecosystems.^70–73^ Electrochemical techniques, known for their rapid response, play a vital role in pesticide detection. For example, the toxic pesticide lindane has been successfully detected using 1D graphitic carbon, such as multi-walled carbon nanotubes (MWCNTs), which significantly enhances sensor sensitivity.^74^ Additionally, molecularly imprinted sensors utilizing graphitic carbon nitride and polyoxometalate nanocomposites have been developed for lindane detection,^75^ showcased the versatility of electrochemical approaches in addressing pesticide contamination concerns.

This study proposed a simple electrochemical surface-enhanced Raman spectroscopy (EC-SERS) method that only requires one “mixing and detection” to detect the neonicotinoid pesticide acetamiprid (AAP). The residues of AAP have become a major concern for fruits and vegetables. In this case, the surface of the screen-printed electrode (SPE) was altered by the application of silver nanoparticles (AgNPs). The technique is a useful tool for keeping track on pesticide residue levels in food samples.^76^ Based on acetylcholinesterase inhibition, an enzymatic electrochemical biosensor was developed for the indirect detection of organophosphates (OPs). Copper nanowires (CuNWs) composited with reduced graphene oxide (rGO) were added to the SPCE in order to improve the biosensor's performance. With its excellent recovery rates and lack of interference, this sensor is helpful for analysing the presence of chlorpyrifos in drinking water and orange juice.^77^ Using impedance electrochemistry spectroscopy, the AgNPs/carbon dots/MWCNTs nanoarchitecture, distributed over a gold-printed electrode surface, demonstrated exceptional electrocatalytic activity for fenitrothion measurement in acetate buffer at pH 4.5, with a detection limit of 0.48 nmol L^−1^. The method was also used to identify fenitrothion pesticide, and other pesticides in orange juices.^78^

A highly sensitive electrochemical aptasensor against chlorpyrifos (CPF) was developed using novel carbon quantum dots-graphite composite ink-based screen-printed electrodes (CQDs/SPEs). With increasing volumes, the produced aptasensor responded well to potato extract that had not been spiked. As a result, the created aptasensor showed a respectable level of application in real agricultural and food samples.^79^ The combination of printed electronics and harmless biopolymeric films produces plant-wearable sensors for decentralized pesticide analysis in precision farming and food safety. This article describes a straightforward process for creating flexible, sustainable sensors that can detect carbendazim and paraquat in food, water, and agricultural samples. The sensors are printed on cellulose acetate (CA) substrates. The analytical performance was evaluated using square wave voltammetry (SWV) and differential pulse voltammetry (DPV) in a linear concentration range of 0.1 to 1.0 μM, with detection limits of 19.8 nM for paraquat and 54.9 nM for carbendazim, respectively. Without being restricted by other pesticides, the versatile and environmentally friendly non-enzymatic plant-wearable sensor can identify carbendazim and paraquat on tomato and lettuce skins as well as in water samples.^80^

#### 5.1.3. Detection of metal ions

Heavy metal ions, which persist in the environment and can lead to adverse effects on human health and ecosystems, require effective removal for protection.^81,82^ Some heavy metals are essential in low quantities but harmful at excessive levels, even leading to cancer or mortality.^83,84^ Electrochemical sensors are key in addressing heavy metal pollution, and modifying electrodes with graphene-based materials significantly enhances their performance. Studies have shown that graphene and its derivatives, when integrated with other materials like bismuth nanoparticles or metal–organic frameworks, greatly enhance sensitivity, selectivity, and detection limits for various heavy metal ions, making them invaluable in environmental and health protection efforts.^85^

The study introduced an electronic tongue (e-tongue) system based on cyclic voltammetry with three different electrodes (pencil graphite, screen printed, and glassy carbon) to detect and analyze heavy metals (cadmium, lead, tin, and nickel) in sunflower edible oil at varying concentrations. The cyclic voltammetry results demonstrated that each electrode (PG, SP, and GC) exhibited different sensitivity levels, with SP showing the highest sensitivity to heavy metals in the oil. Principal component analysis (PCA) was employed for classification, and the results indicated that all three electrodes effectively detected the heavy metals. The variance analysis attributed 98% to SP, 88% to GC, and 84% to PG. The e-tongue system, combined with chemometric analysis, successfully identified cadmium, lead, tin, and nickel at various concentrations in sunflower edible oil, making it a valuable tool for heavy metal detection in food products.^86^

Huang *et al.* reported a disposable sensor that uses square wave anodize stripping voltammetry (SWASV) to detect Pb(ii) and Cd(ii) ions simultaneously. Conjugated mesoporous polymer (CMP) was put onto screen-printed carbon electrodes (SPCE) after bismuth nanoparticles (BiNPs) were electrodeposited therein. This resulted in the CMP/BiNPs/SPCE mixture. The simultaneous measurement of Pb(ii) and Cd(ii) in seafood samples effectively established the usefulness of the technique.^87^ In this study, Jian, *et al.* and his team reported a disposable sensor for measuring lead in genuine tap water, juice, preserved eggs, and tea samples by electrochemically depositing reduced graphene oxide (rGO) on the surface of SPCE.^88^ The results based on rGO-SPCE were reliable and precise when compared to results from graphite furnace atomic absorption spectroscopy (GFAAS), indicating that the disposable sensor has a lot of potential in applications for quick, sensitive, and affordable Pb^2+^ detection in food.

An ion selective membrane containing zinc(ii) phthalocyanine as the ionophore, together with carbon black nanomaterial, were added to the screen-printed electrodes. Recovery values ranging from 90% to 100.5% were observed when apple juice, skim milk, soybean, and coconut water samples were simultaneously tested for Fe^3+^ and K^+^ ions using the newly developed Fe^3+^-selective electrode and K^+^-selective electrode.^89^ A fresh, low-cost disposable porous graphene electrode (P-GE) enhanced with bismuth nanoneedles (nano-BiNDs) is suggested as a “mercury-free” sensor for electrochemical sensing using a smartphone to identify heavy metals. In order to create the P-GE, screen printing was used. On the P-GE, nano-BiNDs were produced using potentiostatic electrodeposition. Pb^2+^ and Cd^2+^ in commercial seaweed products were accurately quantified by the suggested portable sensor, and the results were in line with those of the conventional ICP-OES method.^90^ For the identification of heavy metals, anodic stripping voltammetry (ASV) in conjunction with a screen-printed electrode shows great promise. Using a bismuth/crown ether/Nafion film-modified SPCE, Keawkim *et al.* used sequential injection/anodic stripping voltammetry to measure the lead content in rice.^91^ With a detection limit of 0.11 mg L^−1^ (110 ppb), they discovered a linear detection range for Pb^2+^ of 0.5 to 60 mg L^−1^ (0.5–60 ppm).

## 6. Foodborne pathogen detection

Modified SPEs are employed to find the presence of foodborne pathogens such as *Salmonella*, *E. coli*, and *Listeria*. They can be functionalized with antibodies or DNA probes to selectively capture and quantify specific microorganisms, contributing to food safety. The research done by Bagheryan *et al.*, describes the development of a label-free impedimetric biosensor for the detection of *S. typhimurium* by contrasting two distinct aptasensor fabrication methods.^92^ Electrochemical grafting was found to yield a denser aptamer biorecognition layer, resulting in higher sensitivity, particularly at both low and high *S. typhimurium* concentrations. The aptasensor displayed a linear reaction throughout a large concentration range and exhibited high selectivity for *S. typhimurium*. Importantly, the sensor worked effectively in undiluted real samples, emphasizing its practical applicability. The study's comparison with existing aptasensors highlights its competitive limits of detection and extended dynamic range, making it a useful addition to the field of food analysis and microorganism identification.

Incorporating an innovative approach to DNA detection, the study made by Tsaloglou *et al.* showcases a cost-effective and portable electrochemical methodology designed for remote-sensing applications, in particular resource-limited settings.^19^ By joining recombinase polymerase amplification (RPA) with an electroactive mediator, this portable instrument uses paper-based, throwaway strips with carbon electrodes screen-printed on them. It exhibits precise temperature control, allowing for diverse electrochemical detection methods, and has demonstrated proof-of-concept in screening for diseases, such as tuberculosis, as well as potential applications in monitoring food and water quality. The current sensitivity compares favourably with bench top equipment. This approach holds promise for a wide range of molecular diagnostics, paving the way for accessible, on-site DNA detection beyond laboratory settings.

Wang *et al.* presented a microfabricated electrochemical biosensor intended for the detection of *E. coli* DNA.^93^ This biosensor combines the high specificity of DNA hybridization with the benefits of electrochemical transducers, including portability and affordability, by employing an electrochemical hybridization indicator and screen-printed electrodes. The biosensor's calibration experiments demonstrated a linear response and quantitation capability, with a detection limit of 50 ng mL^−1^ for *E. coli* DNA target. Given its low detection limit, it holds great potential for use in food testing and environmental monitoring. The development of highly specific and sensitive colorimetric and electrochemical DNA biosensors for the quick identification and quantification of *Escherichia coli* in seawater is a significant contribution to the environmental monitoring and water quality assessment.

**Fig. 2:**
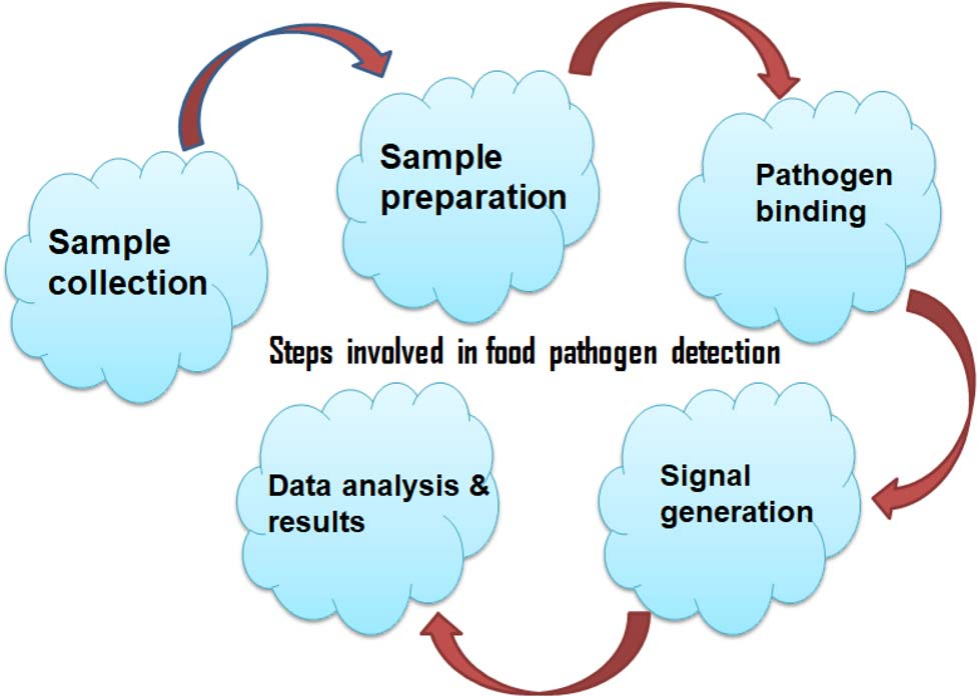
Representation of steps involved in food pathogen detection.

The biosensors developed by Paniel and Baudart showed a high degree of specificity against *E. coli*, successfully discriminating it from other microorganisms.^94^ With the capacity to detect 2 to 20 CFU/100 mL of *E. coli* in seawater, these sensors have potential for in-the-field food and environmental^95^ monitoring and also providing reliable, rapid, and cost-effective results. The study by Flauzino *et al.*,^95^ presents a label-free impedimetric genosensor that may be used to detect traces of pig meat adulteration. This device is crucial in areas where beef is prohibited from containing pork because of allergies, cultural or religious beliefs. The sensor utilizes graphene acid to enhance electrode charge transport properties, enabling the rapid detection of pork residues in beef samples within 45 minutes. This development holds significant promise for creating sensitive and selective point-of-need sensing devices for meat purity monitoring. Incorporating these advancements in screen-printed electrode-based biosensors has proven to be a pivotal step in enhancing food analysis and quality monitoring. The representation of steps involved in food pathogen detection are shown below in Fig. 2.

In this study, screen-printed electrodes (SPEs) were functionalized *via* a facile strategy with surface imprinted polymers (SIPs). The SIP-coated SPEs were used in combination with the heat transfer method (HTM) for the real-time detection of *Escherichia coli*. The sensor was tested in buffer, with a reproducible and sensitive response that attained a limit of detection of 180 CFU mL^−1^. Furthermore, selectivity was assessed by analysing the sensor's response to *C. sakazakii, K. pneumoniae* and *S. aureus* as analogue strains. Finally, the device was successfully used for the detection of *E. coli* in spiked milk as proof-of-application, requiring no additional sample preparation.^96^

Wang *et al.* suggested a polymer sensor based on screen-printed electrodes for the particular detection of *Salmonella typhimurium*.^97^ In order to establish distinct binding cavities, the sensor was built by electropolymerizing dopamine while *Salmonella typhimurium* was present on the electrode surface. With a detection limit of roughly 101 CFU mL^−1^ and a detection time of only 4 min, the sensor showed excellent specificity for *Salmonella typhimurium*. It was also able to distinguish *Salmonella typhimurium* from other common foodborne pathogens like *Escherichia coli* and *Listeria monocytogenes*. Real food samples, such as milk and pork, were used to test the sensor's performance, demonstrating that it is a good fit for the quick and on-site identification of harmful bacteria.

Wang *et al.* reported a detailed review describing the important research progress of DNA-based electrochemical biosensors for the detection of foodborne pathogenic bacteria through four perspectives: representative foodborne pathogens detection using electrochemical approaches, DNA immobilization strategies of aptamers, DNA-based signal amplification strategies used in electrochemical DNA sensors, and functional DNA used in electrochemical DNA sensors.^98^ Finally, perspectives and challenges are presented in this field. This review will contribute to DNA-based electrochemical biosensor in enhancing the nucleic acid signal amplification. All of these investigations together highlight the possibility of on-site, sensitive, and quick detection techniques that greatly aid in guaranteeing food safety.

## 7. Antioxidant capacity assessment

SPEs are used to assess the antioxidant capacity of food samples, such as fruits, vegetables, and beverages. This is crucial for evaluating the potential health benefits and quality of these products. A study on the effects of electrochemical oxidation processes during voltammetric analysis of wine samples, which can obstruct the surface of screen-printed carbon electrodes (SPCE) and result in lower total polyphenols content compared to glassy carbon electrodes. The research explores treatments to remove this fouling layer, highlighting the potential for SPCE reuse in wine analysis, particularly for quality control.^99^

Another research introduces a novel electroanalytical nanostructured sensor for quantifying vitamin C in commercial infant foods. This sensor, SPCEs modified with Au nanoparticles and reduced graphene oxide flakes, offers a low LOD of 0.088 mg L^−1^, making it suitable for quality control labs. Its fast response, ease of use, and disposability make it a valuable tool for assessing vitamin C content in complex food matrices and potentially other compounds relevant in food chemistry.^100^

Using a disposable screen-printed graphene electrode, a straightforward and sensitive electrochemical sensor is displayed for calculating the total vitamin K (K1 and K2) in green vegetables. The study explores parameters affecting sensitivity and demonstrates the sensor's application for quality control of vitamin K1 in natural samples. The method's economic and environmentally friendly nature, as well as its potential for determining other compounds, suggests its utility in food quality analysis and the creation of food composition tables.^101^ There is another research which explores the electrochemical detection of the important antioxidant compound, gallic acid (GA), found in various food and beverage sources. Two detection methods using different devices were employed and validated with actual samples like wine, green tea, apple juice, and serum enhanced with GA. These methods demonstrated specific calibration curves, low detection limits, rapid response times, and excellent sensitivity, which qualifies them for wider uses in determining the antioxidant levels of food, environmental, or clinical samples reported in GA equivalents.^102^

Saribas *et al.* presented a method for measuring gallic acid (GA) at the nanomolar level using modified kaolinite minerals (KNT) and gold nanoparticles (AuNps) on a screen-printed electrode (SPE).^103^ The suggested method's applicability was confirmed through the quantitative analysis of GA in samples of pomegranate juice and black tea. Chakkarapani *et al.*^104^ utilises modified reduced graphene oxide and silver nanoparticles (RGO-Ag NPs) based SPEs to selectively and sensitively determine phenolic substances, such as butylated hydroxyanisole (BHA) and vanillin (VA), which are well-known phenolic compounds frequently utilized as preservatives in various foods and beverages. An electrochemically activated screen-printed boron-doped diamond electrode (aSPBDDE) was developed by Kozak *et al.* for the measurement of the polyphenolic antioxidant curcumin (CCM).^105^ Th prepared sensor was a highly simple, rapid, and sensitive when tested by differential pulse adsorptive stripping voltammetry (DPAdSV) method. The electrode has shown to be a very useful instrument for precisely and accurately determining CCM in food samples directly. A portable technology prototype was created by Ivanova, *et al.*^106^ to measure antioxidant capacity (AOC) potentiometrically. An electrochemical microcell with two identical electrodes with isolated spaces functions as the device's functional unit. The working and reference electrodes were two identical printed electrodes (CSPEs). In a few years, it might be readily modified to serve as a single-use tool for on-site analysis.

## 8. Nutritional analysis

SPEs are employed to measure the concentration of essential nutrients in food products. They can be used to assess vitamins, minerals, and amino acids in different food matrices. An electrocatalytic screen-printed sensor was developed to measure vitamin B1 (thiamine) in food supplements. The sensor, utilizing a cobalt phthalocyanine mediator, allowed the electrochemical conversion of vitamin B1 to an electrochemically active thiolate anion under basic conditions. As a result, selectivity was increased and the detection limit was lowered, resulting in a better analytical response current at an operating potential of 0 V *vs.* Ag/AgCl. The sensor exhibited a linear response within the 0.1 to 20 μg mL^−1^ range, so rendering it appropriate for measuring vitamin B1 in commercial products. The sensor's performance was validated by achieving mean recoveries of 101% for a multivitamin tablet and 93.3% for a multivitamin beverage, suggesting that it may be used to ensure the quality of food supplements and other goods.^107^

Aminoacids: for the purpose of detecting biogenic amines (BAs), which are essential to the safety and quality of food, such as histamine, putrescine, and cadaverine, electrochemical biosensors were created. To construct mono-enzymatic and bi-enzymatic biosensors, these sensors used diamine oxidase (DAO) coupled to magnetic beads (MBs) that were immobilised on different electrodes. Significantly, these biosensors showed outstanding stability over an extended period of time, indicating their suitability for long-term application. They were successfully used to identify BAs in naturally deteriorated and spiked fish, demonstrating their usefulness for quantitative investigation.^108^

A silver/carbon screen-printed electrode (S/C-SPE) was fabricated using polymer-based conductive inks blended with in-house synthesized nanoparticles and investigated for its application in electrochemical analysis. Silver nanoparticles (∼59 nm) and carbon nanoparticles (∼76 nm) were synthesized having zeta potential of <±60 mV and particle morphology were investigated for both the nanoparticles. These nanoparticles were blended with a thermoset epoxy resin to formulate different conductive inks *viz.* silver (Ag) ink, carbon (C) ink and silver/silver chloride (Ag/AgCl) ink. The electrode was applied to determine the level of vitamin C in different fruit juices at the S/C-SPE using a developed voltammetric method and compared with that of standard biochemical method.^109^ Westmacott *et al.* described the development of a novel electrochemical assay for the measurement of water-soluble vitamins in food and pharmaceutical products.^110^ The optimum conditions for the determination of vitamin B_1_ (thiamine), B_2_ (riboflavin) and B_6_ (pyridoxine) in phosphate buffer were established using cyclic voltammetry in conjunction with screen printed carbon electrodes (SPCEs). The method was also applied to a multi-vitamin supplement for the simultaneous determination of thiamine, riboflavin and pyridoxine.

A novel electrochemical sensor was proposed for the simultaneous determination of fat-soluble vitamins (A, D, E, K) using a screen-printed graphene/Nafion electrode (SPGNE). The scanning electron microscopy was used for morphological characterization of the electrode surface. The electrochemical behaviors of fat-soluble vitamins have been studied in a mixture of ethanol and sodium perchlorate monohydrate using square-wave voltammetry (SWV). These developed sensors provided high sensitivity in detection and offer high potential to apply them for the simultaneous determination of fat-soluble vitamins in dietary supplements.^111^ The purpose of this study was to develop an electrochemical sensor for detection of amaranth. The electrochemical sensor based on the modification of a screen-printed electrode *via* polypyrrole nanotubes (PPy NTs/SPE) for detection of amaranth was developed. The preparation of PPy NTs was performed through the pyrrole monomer oxidation with iron(iii) chloride in exposure to methyl orange as structure-guiding agent. Findings exhibited an excellent electrocatalytic activity of as-fabricated sensor for amaranth detection. Moreover, the proposed sensor could practically and successfully determine the amaranth content present in the real food specimens, with acceptable recovery rates.^112^

## 9. Food quality detection

Utilising a recently created biosensor that is built on a screen-printed electrode modified with tyrosinase and single-layer carbon nanotubes (SPE-SWCNT-Ty), hydroxytyrosol (HT) levels in commercial extra virgin olive oils (EVOO) of different kinds and origins were measured. Tyrosinase was rendered to immobile on a screen-printed electrode made of carbon that had been altered to include single-layer carbon nanotubes (SPE-SWCNT-Ty). The results of the study validated the high quality of commercial EVOOs by showing that they contain notable levels of HT. SPE-SWCNT-Ty is a biosensor that shows potential for regular food quality monitoring, especially when evaluating vegetable oils^113^^.^

**Fig. 3:**
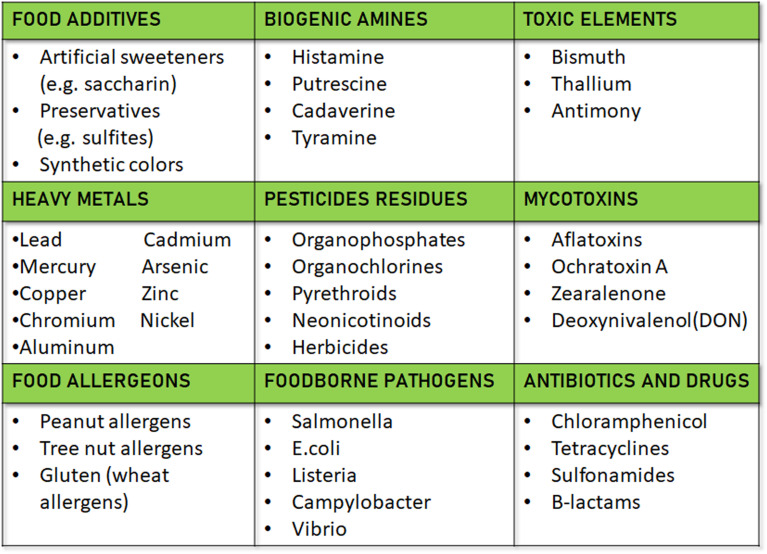
Representation of list of food contaminants detected by SPE.

Duan *et al.*, focused on the rapid detection of sucralose and trichloroacetic acid (TCA), which are halogenated organic compounds, in water bodies.^114^ The Ag/C material is derived from bread waste, contributing to a sustainable circular economy by repurposing food leftovers. The electrochemical process's performance was initially assessed using free chloride ions, followed by the analysis of the target analytes. In response to the increased need for authenticity and quality monitoring, the study offers a technique for differentiating between premium and regular American lager beers. The method uses screen-printed electrodes (SPEs) that are commercially available. These include carbon (SPCE), gold (SPGE), and carbon nanotube (SPEs-CNT) electrodes. The SPEs are combined with chemometric classification techniques like partial least squares regression discriminant analysis (PLS-DA) and soft independent modelling of class analogy (SIMCA). In the validation dataset, the PLS-DA models yielded average scores of 88% for sensitivity, specificity, and precision, whereas the SIMCA models demonstrated average scores of 72%, 82% for specificity, and 80% for precision. Using SPCE in conjunction with PLS-DA, the final setup showed a 94% predictive power, and a single SPCE could analyse up to 35 beers every SPE.^115^ In addition to food quality detection, the presence of heavy metals, toxic elements and food allergens in food materials shows the advantageous aspect of SPE in multidimensional concept and elaborated in Fig. 3 for further discussion.

Investigation done by Cinti *et al.*, proposed a paper-based screen-printed biosensor designed for the purpose of detecting ethanol levels in beer samples.^116^ To detect ethanol, a nanocomposite consisting of Carbon Black (CB) and Prussian blue nanoparticles (PBNPs) was employed as an electrocatalyst. This nanocomposite was utilized to measure hydrogen peroxide generated during the enzymatic reaction between alcohol oxidase (AOx) and ethanol. Following parameter optimization, the biosensor demonstrated the capability to quantify ethanol in various beer types effectively. A single-use screen-printed carbon electrode strip was developed, featuring a surface modification with Pt-MWCNT nanohybrids, for detection of hydrogen peroxide (H_2_O_2_). This sensor strip could effectively measure H_2_O_2_ levels in green tea infusion and pressed tofu, yielding results comparable to those obtained through the ferrous oxidation xylenol orange (FOX) assay and the peroxidase colorimetric method.^117^ In another study, a highly sensitive electrochemical sensor was developed for the precise detection of the cytotoxic food preservative tert-butylhydroquinone (TBHQ) at nanomolar levels in edible oils and water samples which has significant importance in maintaining food quality. The sensor utilized a novel nanocomposite consisting of cupric oxide (CuO) decorated amine-functionalized carbon nanotubes (NH_2_-CNTs).^118^

It achieved an ultra-low detection limit of 3 nM and remarkable sensitivity at 37.7 μA μM^−1^ cm^−2^. The device demonstrated exceptional resistance to interference, stability, reproducibility, and repeatability. Real sample analysis further validated the sensor's practical feasibility, yielding exceptional recovery rates in the range of 95.90% to 104.87% and a maximum relative standard deviation (RSD) of just 2.71%.^119^

## 10. Food safety standards detection

The rapid expansion of the food industry, coupled with the rising need for prolonged shelf life and food conservation, necessitates effective techniques for conveniently monitoring the safety and freshness of food products. This is of paramount significance to consumers, serving to safeguard both their health and finances, and to reduce unnecessary food wastage. The deterioration of food freshness, as observed in items like meat, fish, or poultry, can be discerned through key spoilage indicators originating from the degradation of lipids, proteins, and adenosine triphosphate (ATP).^120^

Xanthine oxidase from *Bacillus pumilus* RL-2d was covalently immobilised onto screen-printed multi-walled carbon nanotubes gold nanoparticle-based electrodes (nano-Au/cMWCNT) to construct an amperometric biosensor for xanthine. With a limit of detection (LOD) of 1.14 nM, the sensor showed a sensitivity of 2388.88 μA cm^−2^ nM^−1^. It was used to evaluate the fish meat's freshness.^121^

A three-dimensional self-supported electrocatalyst was developed by Malhotra *et al.*, incorporating cerium oxide nanocrystals doped with cobalt (CeO_2_–Co) onto tin oxide (SnO_2_) nanorods on a carbon cloth substrate.^122^ This binder-free sensor exhibited excellent sensitivity (3.56 μA μM^−1^), a large linear range (25 nM to 55 mM), and a less detection limit (58 nM) for xanthine (XA) detection. The material was employed in a screen-printed electrode for accurate XA detection in food samples, making it a promising tool for food-safety standards monitoring. This study presents the development of a screen-printed disposable paper device integrated with buckypaper for the detection of phthalates. The device offered a linear detection range from 70 ppm to 15 ppm, with a low detection limit of 12.64 ppm. It demonstrates potential for point-of-care testing in assessing phthalate levels in food safety and quality.^123^

An extensive research carried out by Pennazza *et al.* introduces an innovative voltammetric sensor that interacts with gas and vapor through a screen-printed electrode system immersed in a liquid solution.^124^ The sensor demonstrates acceptable reproducibility and sensitivity in calibration tests with carbon dioxide (CO_2_) and oxygen (O_2_). Additionally, the study showcases its potential for various applications in air quality monitoring, food packaging control, biochemical studies, and biomedical research. Studies presents a straightforward synthesis of gold nanoparticles (AuNPs) decked ultrathin graphitic carbon nitride nanosheets (g-C_3_N_4_) composite using ultrasonication. The resulting g-C_3_N_4_/AuNPs composite is employed to create a highly efficient electrochemical sensor on a screen-printed carbon electrode (SPE) for the sensitive detection of cafeic acid (CA) in numerous food samples. The sensor exhibits rapid response and excellent sensitivity, for regular food quality control and analysis.^125^ This study presents a new electrochemical method using a cobalt phthalocyanine-modified screen-printed carbon electrode (SPCE/CoPc) to investigate myoglobin's scavenging activity against peroxynitrite (PON) in meat extracts. It is a valuable tool for studying the kinetics of oxidative processes involving PON in physiological conditions and assessing the freshness.^126^

SPEs are versatile tools that facilitate precise and cost-effective electrochemical measurements, making them indispensable for food analysis in research, quality control, and compliance with food safety regulations.

## 11. Future directions

**Fig. 4:**
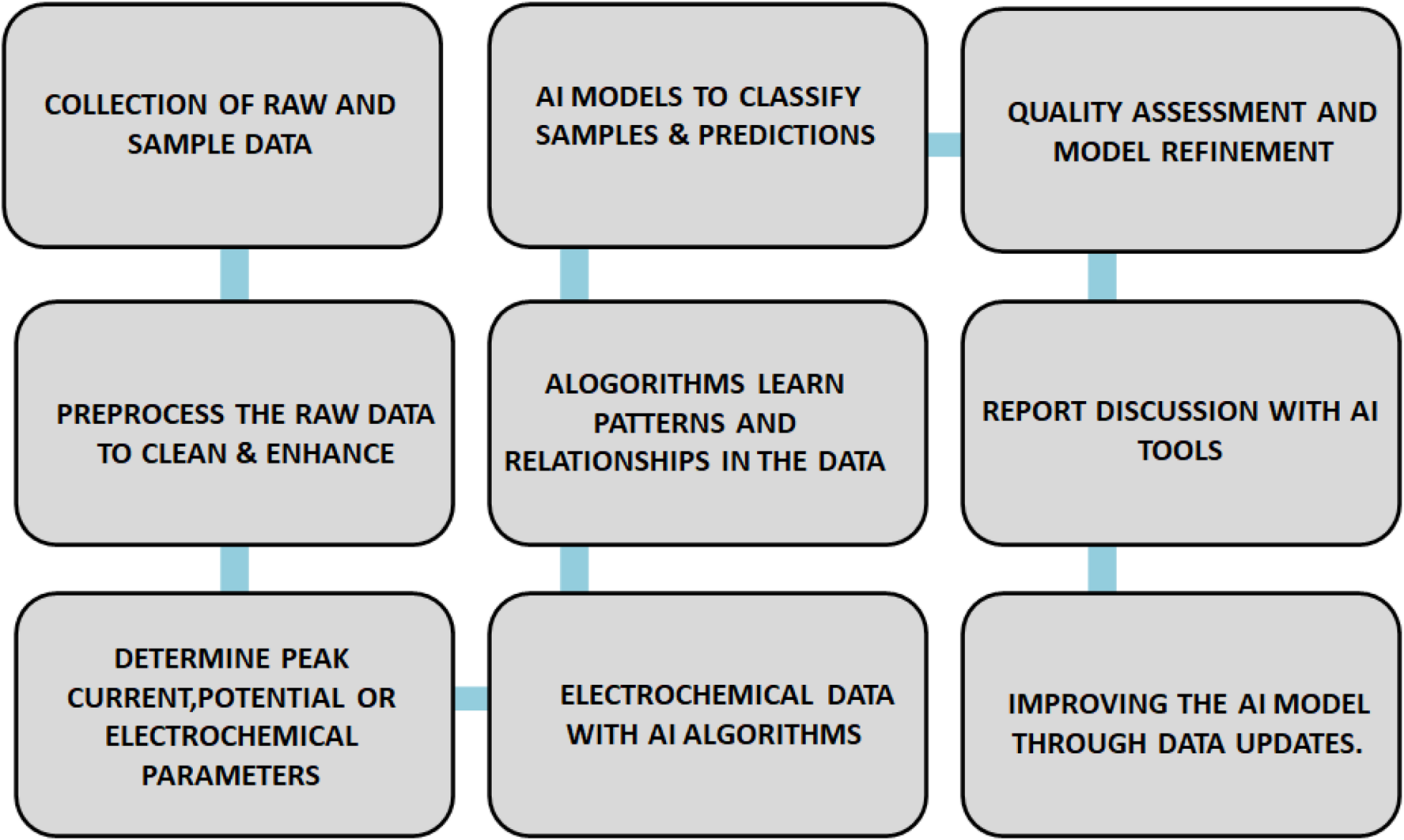
Integration of SPEs with artificial intelligence (AI) for various applications.

Certainly, here are some potential future directions and areas of research and development in the field of screen-printed electrodes (SPEs) in the context of food analysis. Integration of SPEs with AI helps to make predictions on classification, visualization of quality and accuracy of results and its versatile applications is schematically represented in Fig. 4.

### 11.1. Miniaturization and portability

One of the future directions for SPEs is the continued miniaturization and development of portable devices. Researchers can work on creating more compact and user-friendly SPE systems, enabling field testing and on-site analysis. These advancements could have a significant impact on food safety inspections, allowing for real-time monitoring and rapid decision-making.^26^

## 12. Application in wireless sensor and integration with artificial intelligence

Many facets of screen-printed electrode recognition need to be explored in order to progress this sector. A lot of wireless sensor applications involve installing or attaching sensors to the body's surface. Therefore, more investigation is required into the interference and dependability of signals generated by wireless technologies in biological systems. Therefore, extensive clinical trial investigations and coordination and collaboration with medical professionals are necessary for the complete validation of clinical studies. It will assist in elucidating the currently ambiguous link between analytical concentrations and clinical acceptability. Though there is still much to learn about the electronic screen, the full potential of SPEs remains unexplored. Furthermore, multivariate techniques in the expanding fields of chemometrics, machine learning, and artificial intelligence (AI) may be a significant step towards the creation of optimized platforms and their application in multiplexing.^127–130^

In the food business, AI presents an opportunity. Precision farming and numerous other uses in food production and consumption make it indispensable to the upkeep of our food chain. In the food industry, it can also be applied as a quality control method. AI is revolutionizing the way people think about food production, quality, distribution, *etc.*, and the rise of intelligent mobile apps has played a significant role in this shift. Food databases can be efficiently created and analyzed by artificial intelligence. Both customers and employees may benefit from a healthier and more reasonably priced food business as a result. The techniques employed by industry to identify food adulteration are highly costly. Megalingam *et al.*^131^ present a novel approach to food deterioration detection by fusing artificial intelligence, machine learning, and photo categorization (20). In order to identify food that is decaying, they have used AI, deep CNN networks, computer vision, and machine learning techniques like the k clusters approach for color classifications in photos and its HSV values for spoiling detection. This project is completed on the Jupyter notebook platform using the anaconda prompt. Additionally, Iwendi *et al.*^132^ used a network classifier and artificial intelligence to detect and analyze security levels on the internet of things. The study demonstrates a very high accuracy rate for the suggested use. According to this perspective, AI is beneficial in practically every industry today.

### 12.1. Wireless connectivity

**Fig. 5:**
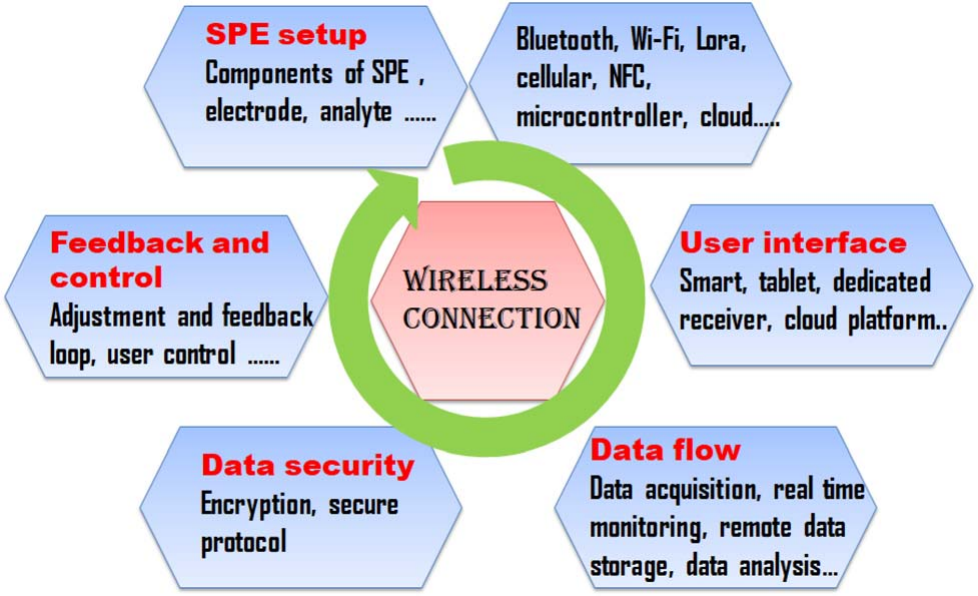
Integration of SPEs with wireless connectivity.

Almalki uutilises drones equipped with Radio Frequency Identification (RFID) sensors to monitor food safety and quality. For safety and security reasons, the proposed model design seeks to wirelessly test the resonance frequency of items dielectric constants from an airborne drone. This work bridges a knowledge gap and presents a novel approach to the Internet of Everything (IoE) in food safety through the use of drones in production, logistics tracking, warehouse management, and product authenticity assessments. Utilizing MATLAB software and simulation results from CST microwave studio, it is confirmed that using drones and RFID to determine the safety and quality of food is a potential and a non-expensive approach.^133^ A simple diagrammatic representation of integration of SPEs with wireless connectivity is represented in Fig. 5.

### 12.2. *In situ* monitoring

Research can focus on the development of SPEs that can be embedded directly in food packaging or storage containers. These sensors would continuously monitor food quality and safety, providing consumers with real-time information on product freshness.^134^

### 12.3. Blockchain and traceability

Combining SPEs with blockchain technology can enhance food traceability and transparency. Each food product could have a unique identifier linked to a blockchain, and SPEs could be used to verify the authenticity and safety of the product at various points in the supply chain.^135^

### 12.4. Personalized nutrition

The development of SPEs that can provide real-time nutritional information based on individual dietary needs and preferences is an exciting future prospect. These sensors could help consumers make more informed choices and promote personalized nutrition.^136^

These future directions hold great promise for the continued advancement of SPE technology in the field of food analysis, contributing to improved food safety, quality, and sustainability. Researchers and industry professionals working together can drive innovation and bring these ideas to fruition, benefiting both the food industry and consumers.

## 13. Advantages and limitations of SPEs

SPEs are advantageous over other methods of electrode production because they allow for simple control over the electrode's composition, thickness, and surface area. Additionally, catalysts can be easily combined with printing ink. Additionally, they provide the opportunity for experimental statistical confirmation of the outcomes even in the case of duplicate electrodes. The fact that they can only be used on flatbeds is their most obvious drawback.^137^ SPEs eliminate the need for pre-treatment or electrode storage and enable the completion of several tests using small amounts of materials and reagents. These electrodes are frequently used for analysis in the food sector, environment, medicine, pharmacy, and agriculture.^138^ SPEs can be generated on demand for analysis, despite the fact that their shapes are known to vary. Additionally, they are available in many shapes as a band, ring, or disc. SPEs are utilised to analyse multiple unknown samples quickly and concurrently in addition to doing calibration. Notwithstanding the aforementioned benefits linked to SPEs, their incompatibility with nonplanar substrates persists as a drawback, hence restricting the fabrication process. Therefore, further work needs to be done on SPEs, which are printed directly onto a variety of flexible and inflexible substrates.^139^

## 14. Conclusion

Screen-printed electrodes (SPEs) have revolutionized the arena of electrochemistry and have become indispensable tool for an extensive range of applications, counting but not limited to environmental monitoring, clinical diagnostics, food analysis, and quality control. SPEs come in various types, each tailored to specific applications, making them versatile and adaptable to diverse research and industrial needs. Their cost-effectiveness and ease of mass production have democratized electrochemical analysis, making it accessible to researchers and professionals.

In the context of food analysis, SPEs have emerged as critical instruments in ensuring the quality, safety, and nutritional content of food products. They play a vital role in detecting contaminants such as toxins, pesticides, and heavy metal ions, providing rapid and cost-effective solutions for food safety assessments. Moreover, SPEs are utilized in the detection of foodborne pathogens, allowing for selective and sensitive quantification of microorganisms that can jeopardize food safety. These sensors find applications not only in laboratories but also in on-site and point-of-care testing, making them crucial in addressing food safety concerns.

SPEs are also instrumental in assessing the antioxidant capacity of food samples, aiding in the evaluation of potential health benefits and quality. They enable the quantification of essential nutrients, vitamins, minerals, amino acids, and other compounds in food matrices, contributing to nutritional analysis and quality control. Additionally, SPEs are used for detecting and distinguishing between various food and beverage products, offering an effective means of food quality detection, thus enhancing consumer confidence and reducing food waste.

Furthermore, as the food industry continues to expand, ensuring food safety and quality is of paramount importance. SPEs are playing an ever-increasing role in the monitoring of food products' safety and freshness. By detecting spoilage indicators arising from the degradation of lipids, proteins, and ATP, they contribute to maintaining the integrity of food products, thereby safeguarding consumers' well-being.

The application of screen-printed electrodes in food analysis has significantly advanced the field, offering precise, efficient, and cost-effective solutions for various food-related challenges. Their versatility, adaptability, and accessibility make SPEs an invaluable resource, enhancing the quality and safety of food products, thereby benefiting both consumers and the food industry. As research and technology continue to evolve, the role of SPEs in food analysis is poised to expand, further contributing to the improvement of food quality and safety standards.

## Conflicts of interest

There are no conflicts to declare.
